# Psychological wellbeing of middle-aged and older queer men in India: A mixed-methods approach

**DOI:** 10.1371/journal.pone.0229893

**Published:** 2020-03-12

**Authors:** Anupam Joya Sharma, Malavika A. Subramanyam

**Affiliations:** Social Epidemiology, Indian Institute of Technology, Gandhinagar, Gujarat, India; University of Sao Paulo Medical School, BRAZIL

## Abstract

Borrowing concepts from public health, we examined the association of several social determinants with the mental health of middle-aged and older queer men in India by combining quantitative and qualitative methodologies. A cross-sectional survey guided by Meyer’s Minority Stress Model was carried out to assess the links between minority stressors (internalized homophobia and degree of closetedness), age-related stressors (ageism and fear of ageing) and psychological wellbeing (loneliness, depressive symptoms and sexual compulsivity) among 207 Indian men (aged 40 years and above) who identified themselves as non-heterosexuals. Results from simple and multivariable linear regression models showed significant positive associations of ageism, internalized homophobia, and fear of ageing with loneliness, even after accounting for sociodemographic and stress mitigating factors. Ageism was not significantly related to depressive symptoms. However, fear of ageing and internalized homophobia was positively associated with depressive symptoms after accounting for covariates. Further, regression models demonstrated a consistent and statistically significant *inverse* association between income and adverse psychological outcomes suggesting the centrality of social class in the lived experience of Indian gay and bisexual men. The qualitative inquiry addressed the same research questions as the quantitative survey through in-depth interviews of thirty middle-aged and older gay and bisexual men in Mumbai. We found that older and midlife gay and bisexual men with higher income (a proxy for social class) found ways to *manage* their masculinities with no discernible adverse psychological outcomes. Depressive symptoms and loneliness in this population made them further vulnerable to excessive sexual impulses, especially in the older queer men who were passing off as heterosexuals. Overall, the theory-driven empirical findings suggest that even in India, where family and friends are social insurance for later life, the issues of ageism and internalized homophobia have the potential to lead to worse mental health outcomes among older queer men.

## Introduction

### Background

Since the 2009 Delhi High Court verdict decriminalizing homosexual acts in India, homosexuality has gained considerable visibility in the country. Queer narratives in the form of activism, books, and newspaper articles have created a space for open discussions about homosexuality. When the Supreme Court re-criminalized it in 2013, the decision provoked agitations from queer activists and allies not just within the country but on a global scale. This led to the historic judgment by the same court decriminalizing homosexuality in 2018. Missing from this recorded history of queer movement in India, the older population of the sexual minority community remained socially invisible in research and the popular media. The global situation mirrors this: Several participants in the few studies of older queer men from the West described gay life as “the province of the young and the beautiful. [1]” Even though we found 42 studies looking at the psychological wellbeing of this population globally, to our knowledge, no study in India has investigated the health of older queer men. This dearth in scholarship might exist because of the invisibility of this population, ignorance about their wellbeing, and the difficulty in recruiting such participants in research due to their need to hide their sexual orientation. Today’s older queer men were raised during the time when homosexuality tended to be medicalized; discussions about sex were limited to private spaces; and ‘homosexual’ was synonymous with ‘sexual deviant’ or ‘pervert’ [2]. In addition, the pervasive homophobic attitudes of a heterosexist society may have discouraged the older queer population from coming out and being counted [3]. In a developing country like India where homosexuality has a long history of criminalization, awareness is limited which likely worsened the situation. An online survey of more than a million queer participants in India, carried out after the Supreme Court decriminalized homosexual activities, showed that almost 40% of the participants were aged 45 years or older, with almost 30% of this group married to women and 20% hiding their gay/bisexual identities from their spouses [4]. Evidence from the US suggests that the degree of “outness” and internalized homophobia could both be linked to lower self-esteem as well as increased psychological distress [5, 6, 7]. Therefore, there is an urgent need to explore these relationships in the Indian context where cultural and social beliefs around homosexuality are barriers to coming out.

### Theoretical framework

Our study is informed by Meyer’s Minority Stress Model [8], which introduced the term ‘minority stress’ to highlight the association between psychological stress and the stigma, discrimination, and violence experienced by oppressed groups. He defined minority stress as (a) *unique* and hence, additive to the general stressors, (b) *chronic*, and, (c) *socially based*, given its birth and growth from social processes and structures. These stressors experienced by minority individuals who have access to few ameliorating factors have several impacts on their mental health [8]. Although the minority stress model has been adopted by many researchers to investigate the relationship between minority stressors and health outcomes, limited studies have explored such relationships in midlife and older sexual minorities. These studies shed light on essential epidemiological phenomena as this population endures age-related stressors in addition to the minority stressors which may combine to affect their mental health [9]. Loss of attractiveness, fear of being rejected within the community due to older age, fears of parental and familial rejection, are health-related stressors in this population [10]. Investigating these links may suggest strategies to address the consequent health impacts. Ageism was shown to have a deleterious impact on mental wellbeing in many studies from the US. Furthermore, in the youth-dominated gay online and offline spaces, the idealized gay body is a “display of masculinity”: lean, muscular, and athletic [11]. Natural ageing brings a decline to these attributes and hence the already feminized social group of older queer men likely gets demasculinized with increasing age. This could lead to a fear of ageing among older queer men risking labelling themselves as “asexual oldie.” This may also give rise to an obsession to look younger if they feed on the consumerist culture and failing to meet the consumerist demands, these men could go through psychological distress [12] which may affect their sexual relationships [13]. Moreover, older queer men could be doubly disadvantaged by the overlap of two minority identities (sexual minority status and older age) which could be further compounded by any disadvantaged identity like low socioeconomic status.

One of the important, insidious, and most proximal stressors that Meyer introduces in his model is ‘internalized homophobia’ defined as “the gay person’s direction of negative social attitude towards the self, leading to a devaluation of the self and resultant internal conflicts and poor self regard” [14]. Internalized homophobia could be a result of any homo-negative experience, abuse, violence or discrimination because of their sexuality and age, and could lead to denial and fear. Thoits [15] refers to this as ‘self stigmatization’ which continues to exist even if the negative events stop occurring or even when one successfully accepts his/her/their sexual identity. Older queer adults of today have grown up in the pre-liberalization India where awareness of homosexuality was limited and where there was no internet for social networking. Their acceptance or non-acceptance of sexual identities may be largely influenced by the cultural, social, religious structures, and family pressures that forced them to conform to the traditional norms of patriarchy and heteronormativity [16]. Thus, historical context is important while conceptualizing internalized homophobia and understanding its impact in India.

Stress ameliorating factors such as social support are emphasised in Meyer’s minority stress model (Meyer, 2003). Social support was found to significantly reduce loneliness [17], was associated with better self-reported general health [18] and lower levels of loneliness and depressive symptoms [19] in similar populations. Social support assumes importance in this population because they face great risk of stigma and discrimination due to their sexual minority status [20] but may lack the support they need in the Indian context. In a family-centric country such as India, social support that the sexual minorities might need could be from their family and closest friends [21]. Moreover, social engagement with people within the sexual minority community and affirmative institutions may also count as ameliorating factors [22]. For instance, the community space *GayBombay* served as a community-based social support in the 1990s, which the older queer men considered as a ‘third space’ other than home and work [23] where they could freely be *themselves* without fear of any discrimination. Additionally, active participation in LGB events and groups was reported to be important in accepting one’s own sexuality [24]. With the emergence of the internet in India after 1995, LGB individuals began interacting with one another and this constant interaction generated a sense of community and built loyalty [16]. A factor that was found to help Chinese sexual minority individuals in ‘coming out’ to family and friends was being in a relationship with a same-sex partner, as it reduced the risk of being rejected by the family [25]. Having a same-sex partner was also found to reduce the risk of loneliness [6, 26]. Thus, the role of a same-sex partner in accepting one’s sexual identity, resisting internalized homophobia or ageing, and avoiding depression and loneliness requires further investigation. Apart from social support, attributes such as optimism and stress resilience have been found to improve the psychological wellbeing of older queer men [27, 14]. Previous studies have also discussed the protective effects of socioeconomic status on several health outcomes in this population.

The present study was guided by the Meyer’s Minority Stress Model [14] and considered stressors hypothesized to be pertinent among older queer men, viz. *ageism* and *fear of ageing*, in addition to the following minority stressors relevant to this population: *internalized homophobia* and *degree of closetedness*.

### The research questions

The broad research questions addressed in this study are:

1 Are age-based discriminatory events, the degree of closetedness, fear of ageing, and internalized homophobia related to depressive symptoms and loneliness in middle-aged and older queer men? We hypothesize that greater age-based discrimination and greater internalized homophobia are related to greater depressive symptoms, and loneliness and lower degrees of closetedness are related to lesser depressive symptoms and loneliness.
2 Do the associations of stressors with the psychological wellbeing persist after accounting for the participants’ sexual orientation, age, relationship status, living arrangements, income, and education?
3 How are the potential stress mitigating factors—participation in LGB events, social support through groups, optimism, and stress resilience—related to depressive symptoms and loneliness?

## Materials and methods

The present study employed a mixed methods approach to address the research questions. While the quantitative study explored how the minority stressors and the stress ameliorating factors were related to the psychological wellbeing of older queer men in India, the qualitative component attempted to understand how the stressors were formed and what their influence was on mental health. We looked at the life histories of the men in our sample and explored their experiences growing up as queer men in the Indian heteronormative society. Following a convergent parallel design [28], a small qualitative preliminary study was conducted in early October 2018 to conceptualize the study and refine the research questions. Later, the qualitative and quantitative data collection and analysis were carried out concurrently from December 2018 through February 2019.

### The preliminary study

A focused group discussion (FGD) with seven older queer men and five in-depth qualitative interviews were conducted in Mumbai. Stewart et.al [29] recommend FGDs as the starting point for designing survey questionnaires. The FGD highlighted the themes of the institution of marriage with a heterosexual partner; the role of family and kin in mental wellbeing; the challenges in coming out; the benefits of queer networks; and, their psychological wellbeing. Many participants discussed their sexual lives, and shared instances suggesting sexual compulsivity. As per several participants such instances were precipitated because of their loneliness or depression. We decided to further investigate these instances and therefore another research question was included in our study:

4 Is there any relationship between loneliness, depressive symptoms and sexual compulsivity in the study population?

We hypothesized that greater loneliness and depressive symptoms would be related to higher sexual compulsivity in this population.

### The quantitative survey

An online survey was carried out among adult Indian cis-men who were attracted to men. The introductory text described the inclusion criteria as men aged 40 years and above and who self-identified themselves as non-heterosexuals. A link to the online Google form (questionnaire) was posted on several LGBTQ focused groups on Facebook. We were unable to identify a specific study site because the link to the form may have been shared widely and participation may not have remained confined to any particular geography. A few of the participants were recruited through the dating app *Grindr* [30] where an advertisement about the study was published. Participants who expressed interest were contacted online and the link was shared. The participants were also requested to circulate the form among their friends and colleagues. Recruiting through several methods helped increase the socioeconomic diversity of our sample.

#### The variables

*Outcomes*. *Loneliness*: Level of the loneliness of the participants was measured through the third version of the University of California Los Angeles (UCLA) Loneliness Scale [31]. The 20 items scale consists of items like “I feel I lack companionship” and the responses are recorded through a four-point Likert Scale ranging from *never* (1) to *always* (4). Nine items were reverse scored. The total loneliness score is the composite score of all item scores. Greater score predicted more loneliness. The scale has been adopted for use among Indian adults by Grover et.al [32]. The scale displayed good reliability in the sample under study, with a Cronbach’s alpha of 0.93 similar to Russel et al [31].

*Depressive symptoms*: To assess depressive symptoms the study used the shorter version of the Geriatric Depression Scale (GDS-15) which consists of 15 items such as “Do you feel happy most of the time”, where the answers were recorded as yes or no. The scale has been validated for older adults by Sheikh & Yesavage [33] and has been widely used. The current study showed strong internal consistency with Cronbach’s alpha of 0.91.

*Sexual Compulsivity*: The Sexual Compulsivity Scale has been adopted for measuring sexual compulsivity in the present study as recommended by George et.al [34]. It is a 10 item scale with items such as “My desires to have sex have disrupted my daily life” and the responses varied from *not at all like me* (1) to *very much like me* (4). Although the scale has not been validated in the India, we found good internal consistency in our sample with a Cronbach’s alpha of 0.89.

*Independent variables*. *Ageism*: An important theme that emerged in the focused group and during the pilot qualitative interviews was discrimination of the older queer men by the younger men in real life as well as dating apps because of the idea of declination of the attractiveness of the body. Based on the reports of age-based discrimination in the preliminary study we developed a measure to assess the ageist discriminatory events in the sample. Two questions were included in the survey. The answers to the question “In the last year, have you ever been discriminated anywhere by someone because of your age?” have been converted to a dichotomous variable of yes or no. The same dichotomy was brought to the answers to the question “Do you think people take/took advantage of you because of your age?” The ageist discriminatory score (ageism) was the sum of the values of the answers to the two questions ranging from 0 to 2. The measure was pretested in 15 older queer men before deployment.

*The degree of closetedness*: Based on the focused group discussion in the preliminary study, we have identified a measure of outness or closetenedness in three levels. The degree of closetedness was assessed by asking about how much they were out about their sexuality. The question had options like closeted (no straight friend or family member knows), partially out, and out to everyone along with an additional option “others” where the participants were granted the liberty to write their own option. Responses like “out to only my best friend”, “out to my mom, but not dad” have been categorized under the partially out category. The degree of closetedness has been measured by assigning a value of 3 to the participants who reported to be closeted, 2 to the participants who are partially out and 1 to the participants who are out to everyone.

*Fear of Aging*: This variable was quantified through a single question, “Do you have any fear of ageing as a gay man?” the binary responses (yes = 1, no = 0).

*Internalized Homophobia*: Internalized Homophobia was assessed through the 9-item Internalized Homophobia Scale developed by John Martin and Laura Dean [8]. Some of the items are, “I have tried to stop being attracted to men in general”, “I feel that being gay/bisexual is a personal shortcoming for me” and the answers ranged from strongly disagree (1) to strongly agree (5). The scale showed good reliability in a sample of 147 homosexual adults in California (Cronbach’s alpha of 0.71 for women and 0.83 for men) [35]. While the scale has not been validated in the Indian population, we found strong internal consistency with Cronbach’s alpha of 0.89 in our sample.

*Covariates*. *Sociodemographic variables*: The sociodemographic variables assessed in the questionnaire were age (in years), sexual orientation (confused, bisexual, and gay), annual income, level of education attainment, relationship status (single and partnered), and place of residence (rural and urban).

*Participation in LGB events* was measured through a single item “Do you actively participate in LGB events?” with a binary response (yes = 1/ no = 0).

*Perceived social support from LGB groups was assessed* using a measure that we developed. It was found in the preliminary study that several participants heavily relied on social networking platforms for social support. We developed and pre-tested a measure using two items, “Are you a part of any LGB group?” (binary, yes = 1, no = 0) and “On a scale of 0 to 10 how beneficial do you think LGB support groups (offline or online) are in keeping you happy?” The composite score of the two items ranging from 0 to 11 was used as the social support measure.

*Optimism*: The Revised Life Orientation Test (LOT-R) developed by Scheier et.al [36] was used to assess optimism in the sample. It is a 10 item scale out of which 3 items measure optimism, 3 items measure pessimism, and 4 items serve as fillers. The response options for all the items range from I agree a lot (1) to I disagree a lot (5). The scale does not have any benchmark for optimism or pessimism and has been adopted in the Indian population [37]. In the current study sample we found a Cronbach’s alpha of 0.68 which is close to the range 0.74–0.78 [38].

*Stress Resilience* was measured using the Brief Resilient Coping Scale (BRCS) developed and validated by Sinclair & Wallston [39] which includes both dispositional (e.g. optimism) and situational (e.g. active problem solving) dimensions of coping. In the present study, the Cronbach’s alpha was found to be 0.77.

#### The qualitative component

The qualitative study explored the experiences of the participants growing up through childhood, their midlife, and then their experiences as older queer men in India.

Thirty gay men aged 40 years and above were recruited through the dating app *Grindr* and through an offline group *Seenagers*, *Mumbai*, an exclusive safe space for older queer men to interact and share their experiences. AJS was introduced to the potential participant pool at *Seenagers* by a key contact, an LGBT activist and an active member of *Seenagers*. AJS also created a *Grindr* profile in September 2018 and used it for recruiting several participants through January 2019. This profile described the study in its main body. *Grindr* members who expressed an interest in the study and were given further details and recruited for interviews. While we reached saturation around our 20^th^ interview, we continued the interviews beyond 20 to increase the diversity of the sample. Our final sample size was 30. In-depth interviews were conducted with all thirty participants at times and locations of their choice such as public cafes or their personal residences. The interviews lasted for an hour to an hour and a half.

### Positionality

For qualitative research, researchers have always acknowledged their position and role of their academic and professional background for a better understanding of the subject experiences. While, eliminating the subjectivity of the researcher in the social sciences is neither desirable nor possible, it gets reflected at least during the interpretation stage where the researcher is bound to add their perspective to the study. Therefore, it is wise to acknowledge the researcher’s subjectivity in such studies.

The motivation to pursue this study is largely influenced by AJS’s emotional involvement and empathetic interaction with the sexual minorities as a part of previous academic work and also his personal life. Witnessing the struggles of several queer individuals, especially the social isolation and alienation experienced by them, led AJS to cultivate an interest in understanding their lives. One of the challenges that AJS faced while conducting the interviews was the difficulty in initial communication and rapport building because of the large age gap between AJS and the researched. The age gap caused many respondents hesitation to open up, especially about their intimate lives, an observation distinctly visible in the interview setting. Few of the participants were reluctant to meet AJS (a young man in his 20s) at public places and were concerned about raising the onlookers’ eyebrows. Many participants, particularly the ones who were highly closeted, were concerned about the benefits and the purpose of their participation. Also, the respondents who lived in Mumbai were more affluent and their luxurious lifestyle and their demeanor of ease in public spaces were very different from the queer individuals that AJS had previously interacted with. Amidst these, several important pieces of information might have gotten lost which could have given a more nuanced insight to the study. But after a few initial interviews the researcher quickly acquainted himself with the new setting and strategized the subsequent interviews in a way the respondents would expect, using body and verbal languages the respondents were familiar with. This strategy came across as respectful, friendly, and reassuring. Almost all interviews were conducted in English.

### Analysis plan

The continuous variables in the survey data were first assessed for normality as per West, Finch and Curran [40]. First, simple linear regression models (Model 1) were fit to estimate the association of each predictor with the outcome variables. Model 2 adjusted for sociodemographic variables and Model 3 additionally included the other covariates. The reference categories for sexual orientation, relationship status, and living arrangement were confused, single, and rural respectively. Multicollinearity between the predictor variables was examined using the Variance Inflation Factor, with correlations ±0.5 to 0.6 (or higher) between predictors identified as problematic [41]. All statistical models were run on STATA software version 12.

The qualitative data were analyzed using a thematic narrative analysis method [42]. Following Merriam [43] and Marshall & Rossman [44] analysis was simultaneously carried out while collecting data. Audio-taped interviews were transcribed verbatim and transcripts were repeatedly checked to avoid mistakes. Major ideas and themes that emerged after repeated reviews were chronicled [43] and indexed as codes. The codes were cross-checked by an external researcher. For respondent validation, follow-up interviews with six participants were conducted to revisit a few of the themes which gave the participants the opportunity to provide feedback and comment on the findings. Finally, an external expert reviewed a description of the research questions, methods, and results, and approved the conclusions drawn.

The quantitative and qualitative studies received equal priority with the strands remaining independent during the analysis while the mixing took place during the interpretation.

### Ethical considerations

The study received ethical clearance from the IIT Gandhinagar Internal Ethics Committee. We obtained written consent from most of the participants. If participants expressed reluctance to sign the written consent form, verbal consent was taken and audio-taped. The online survey participants provided implied consent based on the introductory text inserted at the beginning of the survey. Study details were shared with prospective participants, with explanations provided wherever needed, prior to recruiting them. We stressed that participation was entirely voluntary and that the data collected would remain confidential. The participants were told that there would be no direct benefits to them. All participants’ names used in this study were changed to maintain confidentiality.

## Results

### Quantitative survey

A total of 207 eligible participants completed the survey, of whom 193 (93.23%) provided responses to all questions. Age of participants ranged from 40 to 81 years (mean = 53.51 years, SD = 10.92). A majority of the participants (~60%) were aged 40–50 years, while the rest (~40%) were 51–81 years old. Out of the 207 participants, 158, 38 and 11 self-identified themselves as cis- gay and cis- bisexual and confused queer men respectively. Majority of the participants were well educated with only ~8% of them having education below graduation. In terms of income, almost 60% of the participants earned below Rs 10,00,000 annually, while the rest (~40%) earned well enough to lead a luxurious life in the city. The detailed sociodemographic information about the participants is given in Table 1.

**Table 1:** Sociodemographic characteristics of the middle-aged and older queer men in the quantitative survey (N = 207).

**Age (in years):**	Frequency (Relative frequency %)
40–50	124 (59.90)
51–60	35 (16.91)
61–70	32 (15.46)
71 +	16 (7.73)
**Self-reported Sexual Orientation:**	
Gay	158 (76.33)
Bisexual	38 (18.36)
Unsure	11 (5.31)
**Self-identified Current Relationship Status:**	
Currently single	130 (62.80)
Partnered	77 (37.20)
**Living Arrangement:**	
Rural	12 (5.80)
Urban	195 (94.21)
**Education:**	
High school and below	5 (2.42)
Completed High school and some higher secondary education	11 (5.31)
Completed Higher secondary Education	87 (42.03)
Graduate (college education)	94 (45.41)
Graduate and above (including PhDs and other professional degrees/diploma)	10 (4.83)
**Annual Income in Indian Rupees (1 USD = approx. Rs 70):**	
0 to 300, 000	64 (30.92)
300,000 to 10, 00,000	62 (29.95)
10,00,000 to 20,00,000	41 (19.81)
20,00,000 to 50,00,000	24 (11.59)
50,00,000 and above	16 (7.73)

#### Loneliness

Ageism was positively associated with loneliness in the fully adjusted multivariable linear regression model (b = 2.85, p<0.01). Reports of loneliness were higher among those who reported a fear of ageing (b = 7.55, p<0.001). Internalized homophobia was positively associated with higher levels of loneliness (b = 0.63, p<0.001). Annual income was mostly inversely associated with loneliness (see Tables 2–5). None of the other sociodemographic predictors were significantly associated with loneliness. Model 3 showed inverse associations of LGB participation, optimism, and stress resilience with the outcome loneliness (see Tables 2–5).

**Table 2:** Association (regression coefficient, 95% confidence intervals) of ageism with depressive symptoms and loneliness among middle-aged and older queer men in India.

	Depressive symptoms			Loneliness		
Predictor	Model 1	Model 2	Model 3	Model 1	Model 2	Model 3
**Ageism**	0.766 (-1.33, 1.67)	0.798 (-0.030, 1.625)	0.772 (-0.008, 1.552)	3.213** (0.92, 5.50)	2.984** (0.876, 5.093)	2.853** (0.903, 4.803)
**Age**		0.010 (-0.048, 0.067)	0.012 (-0.041, 0.065)		0.015 (-0.132, 0.161)	0.040 (-0.093, 0.173)
**Sexual orientation**						
Bisexual		-2.343 (-5.418, 0.731)	-1.212 (-4.037, 1.613)		-3.493 (-11.331, 4.344)	-1.930 (-8.992, 5.132)
Gay		-2.591 (-5.337, 0.155)	-0.972 (-3.544, 1.599)		-5.925 (-12.924, 1.075)	-0.990 (-7.418, 5.438)
**Relationship status**						
Partnered		0.175 (-1.152, 1.501)	-0.215 (-1.502, 1.073)		-1.193 (-4.575, 2.190)	-2.219 (-5.438, 0.999)
**Educational level**		-0.818 (-1.774, 0.138)	-0.655 (-1.580, 0.270)		0.411 (-2.023, 2.847)	1.356 (-0.957, 3.668)
**Living arrangement**						
Urban		-2.153 (-4.989, 0.683)	-0.541(-3.131, 2.050)		-3.829 (-11.058, 3.401)	-0.524 (-7.001, 5.953)
**Annual income**		-1.292*** (-1.841, -0.742)	-0.968*** (-1.484, -0.450)		-3.981*** (-5.381, -2.580)	-3.070*** (-4.363, -1.776)
**Participation in LGB events**			-2.098** (-3.493, -0.702)			-6.922*** (-10.410, -3.434)
**Optimism**			-0.307*** (-0.453, -0.161)			-0.716*** (-1.081, -0.352)
**Stress resilience**			-0.337*** (-0.535, -0.138)			-0.682** (-1.178, -0.187)
**Perceived social support**			-0.041 (-0.240, 0.158)			0.131 (-0.367, 0.628)
N	200	200	188	200	200	188

* p<0.05,

** p<0.01,

*** p<0.001

Model 1: Unadjusted model

Model 2: Adjusted for age, sexual orientation, relationship status, educational attainment, living arrangement, and annual income

Model 3: Fully adjusted model (additionally adjusted for participation in LGB events, optimism, stress resilience, and perceived social support)

**Table 3:** Association (regression coefficient, 95% confidence interval) of degree of closetedness with loneliness and depressive symptoms among middle-aged and older queer men in India.

	Loneliness			Depressive symptoms		
Predictor	Model 1	Model 2	Model 3	Model 1	Model 2	Model 3
**Closetedness**	6.300*** (4.125, 8.475)	2.519* (0.452, 4.585)	1.320 (-0.884, 3.524)	1.882*** (0.997, 2.767)	0.589 (-0.295, 1.474)	0.043 (-0.861, 0.947)
**Age**		-0.024 (-0.143, 0.096)	0.002 (-0.120, 0.116)		-0.010 (-0.061, 0.042)	-0.007 (-0.056, 0.041)
**Sexual orientation**						
Bisexual		-3.942 (-10.558, 2.674)	-2.742 (-9.210, 3.726)		-3.086* (-5.916, -0.256)	-2.137 (-4.791, 0.516)
Gay		-0.301 (-6.384, 5.782)	1.008 (-4.925, 6.942)		-0.852 (-3.454, 1.750)	-0.259 (-2.694, 2.175)
**Relationship status**						
Partnered		-1.963 (-4.770, 0.844)	-2.785 (-5.659, 0.089)		-0.123 (-1.324, 1.078)	-0.689 (-1.868, 0.490)
**Educational level**		1.028 (-0.896, 2.952)	0.780 (-1.254, 2.814)		0.137 (-0.686, 0.960)	-0.317 (-1.152, 0.517)
**Living arrangement**						
Urban		1.892 (-3.882, 7.665)	1.338 (-4.600, 7.276)		0.844 (-1.626, 3.314)	-0.041 (-2.477, 2.395)
**Annual income**		-2.927*** (-4.144, -1.709)	-2.436*** (-3.644, -1.228)		-0.980*** (-1.501, -0.460)	-0.747** (-1.244, -0.252)
**Participation in LGB events**			-3.502* (-6.932, -0.073)			-1.026 (-2.433, 0.381)
**Optimism**			-0.509** (-0.854, -0.164)			-0.216** (-0.358, -0.075)
**Stress resilience**			-0.443 (-0.888, 0.002)			-0.323*** (-0.506, -0.141)
**Perceived social support**			-0.013 (-0.465, 0.439)			-0.055 (-0.240, 0.131)
**Internalized homophobia**		0.731 (0.533, 0.930)	0.593*** (0.382, 0.806)		0.263 (0.178, 0.348)	0.234*** (0.147, 0.322)
N	207	207	193	207	207	193

* p<0.05,

** p<0.01,

*** p<0.001

Model 1: Unadjusted model

Model 2: Adjusted for age, sexual orientation, relationship status, educational attainment, living arrangement, annual income, and internalized homophobia

Model 3: Fully adjusted model (additionally adjusted for participation in LGB events, optimism, stress resilience, and perceived social support)

**Table 4:** Association (regression coefficient, 95% confidence intervals) of fear of ageing with depressive symptoms and loneliness among middle-aged and older queer men in India.

	Depressive symptoms			Loneliness		
Predictor	Model 1	Model 2	Model 3	Model 1	Model 2	Model 3
**Fear of ageing**	3.730*** (2.492, 4.968)	3.221*** (2.002, 4.441)	2.451*** (1.205, 3.697)	9.815*** (6.645, 12.986)	8.230*** (5.110, 11.350)	7.556*** (4.515, 10.518)
**Age**		0.001 (-0.053, 0.055)	0.005 (-0.047, 0.057)		-0.003 (-0.142, 0.136)	0.027 (-0.099, 0.154)
**Sexual orientation**						
Bisexual		-1.755 (-4.665, 1.155)	-1.140 (-3.897, 1.618)		-2.280 (-9.726, 5.165)	-2.229 (-8.961, 4.503)
Gay		-2.117 (-4.708, 0.475)	-0.819 (-3.316, 1.677)		-4.638 (-11.268, 1.993)	-0.307 (-6.402, 5.789)
**Relationship status**						
Partnered		0.630 (-0.633, 1.893)	0.135 (-1.131, 1.401)		-0.169 (-3.401, 3.063)	-1.385 (-4.476, 1.707)
**Educational level**		-0.812 (-1.720, 0.095)	-0.671 (-1.578, 0.236)		0.344 (-1.978, 2.666)	1.147 (-1.068, 3.362)
**Living arrangement**						
Urban		-2.144 (-4.812, 0.524)	-0.779 (-3.293, 1.735)		-3.889 (-10.716, 2.938)	-1.355 (-7.494, 4.783)
**Annual income**		-1.777*** (-1.696, -0.658)	-0.905*** (-1.408, -0.403)		-3.684*** (-5.011, -2.357)	-2.862*** (-4.089, -1.635)
**Participation in LGB events**			-2.048** (-3.403, -0.694)			-6.905*** (-10.212, -3.598)
**Optimism**			-0.216** (-0.365, -0.067)			-0.461* (-0.826, -0.098)
**Stress resilience**			-0.318** (-0.511, -0.125)			-0.605* (-1.076, -0.133)
**Perceived social support**			-0.024 (-0.219, 0.171)			0.144 (-0.332, 0.619)
**Ageism**		0.318 (-0.479, 1.116)	0.380 (-0.401, 1.160)		1.802 (-0.238, 3.843)	1.697 (-0.210, 3.603)
N	202	197	185	202	197	185

* p<0.05,

** p<0.01,

*** p<0.001

Model 1: Unadjusted model

Model 2: Adjusted for age, sexual orientation, relationship status, educational attainment, living arrangement, annual income, and ageism

Model 3: Fully adjusted model (additionally adjusted for participation in LGB events, optimism, stress resilience, and perceived social support)

**Table 5:** Association (regression coefficient, 95% confidence intervals) of internalized homophobia with depressive symptoms and loneliness among middle-aged and older queer men in India.

	Depressive symptoms			Loneliness		
Predictor	Model 1	Model 2	Model 3	Model 1	Model 2	Model 3
**Internalized homophobia**	0.275*** (0.201, 0.349)	0.283*** (0.203, 0.362)	0.236*** (0.154, 0.318)	0.824*** (0.645, 1.003)	0.817*** (0.630, 1.005)	0.636*** (0.436, 0.867)
**Age**		-0.008 (-0.059, 0.043)	-0.007 (-0.055, 0.041)		-0.016 (-0.137, 0.105)	0.002 (-0.116, 0.119)
**Sexual orientation**						
Bisexual		-3.194* (-6.025, -0.364)	-2.148 (-4.785, 0.488)		-4.403 (-11.087, 2.280)	-3.078 (-9.528, 3.372)
Gay		-1.024 (-3.618, 1.570)	-0.266 (-2.690, 2.158)		-1.037 (-7.162, 5.089)	0.808 (-5.122, 6.738)
**Relationship status**						
Partnered		-0.052 (-1.251, 1.146)	-0.683 (-1.853, 0.486)		-1.661 (-4.491, 1.169)	-2.604 (-5.465, 0.257)
**Educational level**		0.258 (-0.546, 1.062)	-0.309 (-1.124, 0.506)		1.543 (-0.356, 3.442)	1.026 (-0.968, 2.020)
**Living arrangement**						
Urban		0.903 (-1.569, 3.376)	0.045 (-2.473, 2.383)		2.145 (-3.694, 7.984)	1.219 (-4.722, 7.160)
**Annual income**		-1.046*** (-1.559, -0.534)	-0.751*** (-1.240, -0.264)		-3.208*** (-4.418, -1.998)	-2.551*** (-3.746, -1.357)
**Participation in LGB events**			-1.044 (-2.392, 0.303)			-4.073* (-7.371, -0.776)
**Optimism**			-0.215** (-0.354, -0.076)			-0.471** (-0.811, -0.132)
**Stress resilience**			-0.324*** (-0.506, -0.142)			-0.457* (-0.902, -0.012)
**Perceived social support**			-0.054 (-0.289, 0.130)			0.005 (-0.446, 0.457)
N	207	207	193	207	207	193

* p<0.05,

** p<0.01,

*** p<0.001

Model 1: Unadjusted model

Model 2: Adjusted for age, sexual orientation, relationship status, educational attainment, living arrangement, and annual income

Model 3: Fully adjusted model (additionally adjusted for participation in LGB events, optimism, stress resilience, and perceived social support)

#### Depressive symptoms

Ageism was not significantly associated with depressive symptoms in any model. Internalized homophobia was positively associated with depressive symptoms (b = 0.23, p<0.001) in Model 3. Annual income showed an inverse association with depressive symptoms independent of other variables (see Tables 2–5). Greater LGB participation, higher optimism, and higher stress resilience were associated with lesser depressive symptoms (see Tables 2–5).

#### Sexual compulsivity

In the fully adjusted models, loneliness and depressive symptoms were positively associated with sexual compulsivity (b = 0.32, p<0.01 and b = 88, p<0.001, respectively). Only annual income among the sociodemographic factors was associated with sexual compulsivity with a regression coefficient of b = 1.07 (p<0.01) in the fully-adjusted models for both the predictors loneliness and depressive symptoms. No stress ameliorating factor was significantly associated with sexual compulsivity (see Tables 6 and 7).

**Table 6:** Association (regression coefficient, 95% confidence intervals) of depressive symptoms with sexual compulsivity among middle-aged and older queer men in India.

Predictor	Model 1	Model 2	Model 3
**Depressive symptoms**	0.775*** (0.604, 0.946)	0.563*** (0.304, 0.822)	0.638*** (0.346, 0.929)
**Age**		0.002 (-0.072, 0.075)	0.003 (-0.075, 0.081)
**Sexual orientation**			
Bisexual		3.153 (-0.965, 7.272)	3.325 (-0.960, 7.611)
Gay		2.302 (-1.402, 6.006)	2.354 (-1.558, 6.267)
**Relationship status**			
Partnered		-0.831 (-2.565, 0.904)	-0.234 (-2.144, 1.676)
**Educational level**		-0.441 (-1.601, 0.720)	-0.079 (-1.416, 1.257)
**Living arrangement**			
Urban		-2.217 (-5.729, 1.295)	-0.985 (-4.921, 2.951)
**Annual income**		1.071** (0.283, 1.860)	1.018* (0.186, 1.850)
**Participation in LGB events**			0.214 (-1.978, 2.405)
**Optimism**			-0.066 (-0.298, 0.165)
**Stress resilience**			0.097 (-0.209, 0.402)
**Perceived social support**			0.146 (-0.154, 0.446)
**Loneliness**		0.163 (0.058, 0.267)	0.154** (0.037, 0.272)
N	207	207	193

* p<0.05,

** p<0.01,

*** p<0.001

Model 1: Unadjusted model

Model 2: Adjusted for age, sexual orientation, relationship status, educational attainment, living arrangement, annual income, and loneliness

Model 3: Fully adjusted model (additionally adjusted for participation in LGB events, optimism, stress resilience, and perceived social support)

**Table 7:** Association (regression coefficient, 95% confidence intervals) of loneliness with sexual compulsivity among middle-aged and older queer men in India.

Predictor	Model 1	Model 2	Model 3
**Loneliness**	0.290*** (0.221, 0.358)	0.163** (0.058, 0.267)	0.155** (0.037, 0.272)
**Age**		0.002 (-0.072, 0.075)	0.003 (-0.075, 0.081)
**Sexual orientation**			
Bisexual		3.153 (-0.965, 7.272)	3.325 (-0.960, 7.611)
Gay		2.302 (-1.402, 6.006)	2.354 (-1.558, 6.266)
**Relationship status**			
Partnered		-0.831 (-2.565, 0.904)	-0.234 (-2.144, 1.676)
**Educational level**		-0.441 (-1.601, 0.720)	-0.079 (-1.416, 1.257)
**Living arrangement**			
Urban		-2.217 (-5.729, 1.295)	-0.985 (-4.921, 2.951)
**Annual income**		1.071** (0.283, 1.860)	1.018* (0.186, 1.850)
**Participation in LGB events**			0.214 (-1.978, 2.405)
**Optimism**			-0.066 (-0.298, 0.165)
**Stress resilience**			0.097 (-0.209, 0.402)
**Perceived social support**			0.146 (-0.154, 0.446)
**Depressive symptoms**		0.563 (0.304, 0.822)	0.638*** (0.346, 0.929)
N	207	207	193

* p<0.05,

** p<0.01,

*** p<0.001

Model 1: Unadjusted model

Model 2: Adjusted for age, sexual orientation, relationship status, educational attainment, living arrangement, annual income, and depressive symptoms

Model 3: Fully adjusted model (additionally adjusted for participation in LGB events, optimism, stress resilience, and perceived social support)

### Qualitative results

Thirty respondents (Table 8) shared their life experiences of growing up as queer men in India and their experiences with ageism, loneliness, and ageing in general. Several intersecting forms of structural oppression emerged from the interviews. While not every interview provided evidence of all the themes, at least one theme was identified in every interview. Experiences of discrimination due to age or sexual minority status were not universally reported. However, they formed the key components of several interviews and were highlighted in the narratives.

**Table 8:** Distribution (frequency) of the sociodemographic characteristics of the respondents (N = 30) in the qualitative study.

**Age (in years):**	Frequency (Relative frequencies %)
40–50	12 (40)
51–60	11 (36.67)
61–70	5 (16.67)
71 +	2 (6.66)
**Self-reported Sexual Orientation:**	
Gay	18 (60)
Bisexual	8 (2.67)
Unsure	4 (13.33)
**Self-identified Current relationship status:**	
Currently single	12 (40)
In a relationship with another man	7 (2.33)
Bisexual, currently in relation with another woman	5 (16.67)
In a heterosexual marriage	6 (20)
**Living Arrangement:**	
Living alone	5 (16.67)
Living with partner (men)	5 (16.67)
Living with partner (women)	8 (26.67)
Living with family or friends	12 (40)
**Education:**	
High school and below	1 (3.33)
Completed High school and some higher secondary education	2 (6.67)
Completed Higher secondary education	2 (6.67)
Graduate (college education)	17 (56.67)
Graduate and above (including PhDs and other professional degrees/diploma)	8 (26.66)
**Annual Income in Indian Rupees (1 USD = approx. Rs 70):**	
0 to 300, 000	7 (23.33)
300,000 to 10, 00,000	6 (20)
10,00,000 to 20,00,000	9 (30)
20,00,000 to 50,00,000	3 (10)
50,00,000 and above	5 (16.67)

We identified the following salient themes:

#### Shaping of internalized homophobia and its consequences

Many of the participants (23/30) talked about their struggles with their sexual identity in their early lives. The heteronormative space they grew up in convinced many of them to believe that homosexuality was a sin or morally wrong. They shared how they had changed the way they behaved in order to fit the norms of society, thus pretending to lead a heterosexual life. Several (16/30) of the respondents shared stories of how living a life which alienated them from themselves deeply impacted their mental state. The following quote from Sumit (42y) illustrates such challenges as he shared details about his identity as a young bisexual man.

*I was extremely uncomfortable, partly because back then it was not accepted socially and culturally. There was no political correctness, and homophobia was extreme, which made me more uncomfortable with my sexuality. I realized that I am gay when I was in 11^th^ but could not accept it. There was no support system. I considered it to be socially and politically incorrect. My family is Catholic which added religious complications in accepting my identity. […] My personal background of belonging to a reasonably conservative family where there was Catholic preaching about monogamy has somehow influenced me to become hesitant having someone in my life or in bed at that time. I was frightened of my sexuality. I cursed myself for the sin. I lost almost 29 years of my gay life by the time I came out at the age of 38*.

Even though Sumit realized he was different from his peers, he refrained from exploring his sexuality because of the heterosexual socialization around him. It prevented him from leading an authentic life where his desires mirrored his sexuality. Sumit’s narrative provides a rich example of how the heteronormative structure of the society where he was raised led to the development of internalized homophobia. This is evident in the manner in which he not only refrained from disclosing his sexuality to others but also failed to accept it himself. Yogi (50y) had a similar story. He said

It was suffocating for me to suppress my sexual desires during my childhood because of society. It made me have a distaste for sexual things. I remained isolated sexually for years and suppressed these feelings. I have been married for 16 years now and I don’t know what I want in my life in terms of sexual desires. I have a lovely wife who understands me and is comfortable with my sexuality but life is far more abstract and complex now. Many times, these thoughts overwhelmed me and I was stressed. I think, “Okay, it’s not like I am not having a good time. Maybe it is destined to be like this …” We gave each other a good time and we moved on. I sometimes wonder what life would have been otherwise but then I stick to reality, the ship has already sailed taking these pains and I shut myself. I don’t give myself the space to burden my thoughts.

By suppressing his sexual desires and the exploration of his sexuality Yogi not only faced emotional consequences but also maintained a state of confusion within him. Even though he had the support of his loving wife, his life had become far more unpredictable in terms of his sexual desires. Internalized homophobia appeared to generate overwhelming thoughts about leading a different life and left the person with a gnawing feeling when he realized that it was too late to change his life.

#### Negotiating ageist discourse

Almost all the participants (28/30) reported feeling several bodily changes once they hit forty. They claimed that they became ‘unattractive’ and failed to meet certain norms expected in online dating spaces like *Grindr* which idealized young age and bodies. Several participants reported facing abuse from younger *Grindr* users when the participants approached them with a “simple and gentle hello.” Apart from abuse there was unresponsiveness from younger men which the respondents perceived as ageism. Sumit (42y) said

*Till around 38, Grindr was never an issue because I got the attention from people, a huge chunk of them, but that soon stopped when I reached 40. After realising this age prejudice, I stopped putting up my age on my profile and displayed just my picture, where I look much younger. But as I stopped colouring my hair I stopped receiving responses from people who were consistently talking to me. The rejection in the apps makes me stressed. There is no emotional and sexual attention from anyone out there. This rather keeps haunting me*.

Such reports suggest that the ageist discourse in *Grindr* neglects a wide section of the population, rendering them invisible and creating a sense of isolation. The unresponsiveness, rejection, and abuse that the respondents faced stressed them to a large extent. For Kirti (62y), *Grindr* was the only way of getting in touch with people from the community as he was closeted. It was a space for him to get emotionally and sexually connected with people. But several discriminatory events made him delete the app and hibernate from that space. He was unhappy being gay and being old. While many (14/30) like Kirti failed to fight discrimination and embraced stress and depression, the others considered such a discourse as a minor matter. A positive attitude was cited as a key attribute in negotiating the ageist discourse prevalent in the dating app space. Shekhar (61y) shared,

*Ageing is a natural process. Acceptance or rejection matters on your own attitude. Although I try to maintain my body and sanity after the 50s because these naturally decline, the ultimate goal is to stay fit and accept things the way they are*.

Proactively dealing with one’s control over negativities in life, positively accepting and acknowledging the changes pertaining to the body, and thus ageism on *Grindr* or offline, were cited as having helped many participants stay happy.

#### Addressing loneliness and depression

More than sixty per cent of the respondents reported feeling lonely. A feeling of alienation, particularly from the heterosexual universe, was very common. Each individual had varying stories of resisting isolation, loneliness, and depression. Mohit (52y) shared how his way of living alone for quite a long period of time helped him adapt to the loneliness. He said

I do feel lonely at times. But given that I have lived alone for so long, there is a part of me that loves solitude. Solitude is different from loneliness. You can be with your friends and still feel lonely. On the other hand, you can be lonely and still be content. It’s not like that I don’t have those moments (of loneliness), but it’s quite lesser than what many others experience.

Whenever he got caught in the moments of loneliness, he preferred reading a book in his living room and spent hours doing so. He remained calm and content. A similar positive take was shared by Yogi (50y), who said

*My sense of empathy and compassion has been strengthened* (with time). *I write*. *I am lively within*. *I see a confluence of many things in me*. *Optimism is the key*. *Mere saath bura bohot hua hain but uske beech khamosh khushiya bhi hain* (many bad things have happened to me, but amidst those, there was silent happiness).*’*

Mohit and Yogi, similar to many other respondents, showed optimism and adaptation in response to the situation of loneliness. It was evident that Mohit’s ways of living in solitude in the past helped him tackle loneliness in the present. Apart from the personal skills that many (20/30) had developed to negotiate with loneliness, community-based support emerged as an effective buffer to loneliness and depression among the older queer men in Mumbai. For instance, Pratik (60y), shared

It’s always nice to meet people of your age. I believe social spaces like ‘Seenagers’ is trying hard to give us the space to come together and discuss our lives, our struggles as well as happiness.

Several respondents pointed to the immediate need for such safe spaces to connect to peers and discuss issues pertaining to growing old as queer men. A few respondents talked about the importance of physical spaces created by the LGBT community in the form of social gatherings, such as *Seenagers*, where a few of the older queer men meet over a cup of coffee at some public space and discuss their lives. They said that it was soothing to hear stories from peers on how they navigated their lives. Most of them (~90%) discussed how such support groups have helped them in coping with societal stigma, depression, and reducing the sense of loneliness.

#### The role of (hetero-homo) companionship at later life

Although the nine respondents who were in long term relationships with men reported being content with their life and ageing, not all of the fourteen who were single reported being lonely or discontent either. Having a partner (same-sex) had helped many of them be more confident about their sexual identity and thus in coming out to their friends and family. This was evident in what Soumesh (67y) shared,

*My relationship with my partner has made me confident in accepting my sexuality. Being in a relationship always comes with its perks. The family takes it little light when you come out introducing your partner rather than making it a solo act*.

This suggests that while coming out may risk rejection from the family, introducing the partner as a friend and then slowly merging him into the familial space “poses very little threat to the parents’ relationship with their son or that son’s relationship with his lover.[25]” Soumesh also said,

*I have never ‘come out’ to my family. They just understood*.

The interviews suggested that intimate same-sex relationships help in crystallizing the sense of self as a sexual minority individual. While a few (12/30) of the participants considered same-sex relationships to be social and emotional security at an older age, the remaining eighteen firmly held the view that one need not have a partner to lead a content life. Mohit (52y), said

*Relationship is not a mandatory factor to be happy. I don’t know if I am an outlier in this respect. Maybe because my social support system, my straight friend circle is fairly active and large and sometimes I feel it has compensated for this vacuum without my knowing. I don’t believe in ‘we will grow old together’. It is little illusionary. When you live a major component of your life yourself, you live your life on your own terms and the space for somebody else to come in and you accepting is difficult. I don’t date guys often. The natural urge is quite low. When I look at my peers I feel that they look at their relationship as a safety net for old age. It’s sort of absurd for me. There is no legal framework and also in case of heterosexual marriages, there are so many failed marriages. That’s one part of the philosophy where I am not driven towards a relationship*.

The lack of a legal framework for homosexual marriages that repelled Mohit echoes the important socio-cultural context of marriage in India, widely considered as a “duty” [45] and a form of physical-material security [46]. Ruth Vanita writes, “In India, most people have been, and many continue to be, married off at a very young age. Hence exclusive same-sex relationships are rare” [21]. Because of the centrality of marriage, many of the respondents faced pressure for marriage with opposite-sex partners at a very young age. For instance, Kumar (42y), was forced to marry a woman a few years ago. His marriage broke off within two years traumatizing him and his family. His parents’ continual insistence on re-marriage and the fact that he had not come out to his family or friends led him to feel imprisoned. This was reflected in his narrative

*They will not accept me. I will lose all the inheritance and I am not financially independent to support my living in a city like Mumbai. I look for a relationship with someone like-minded, who could understand me and support me*.

Married to women, Prasen and Ashutosh were living a dual life. Committing fully to such relationships was viewed by a few respondents (4/6 married to women respondents) as the right thing to do, although their urges remained unsatisfied. As described by Prasen,

*When she is with me, I devote my entirety to her. I don’t use dating apps, and would not go out for a ‘hook-up’. I become her husband, the way she wants. I have more sexual desires for guys than girls, but at the same time satisfy my wife, sexually, is my responsibility and duty. I have different fantasies that my wife could never fulfil and I could never let them go*.

While several were contented with their marriages to women, many reported feeling suffocated. Ashutosh shared,

*I try to keep my libido under control*. *I love my wife wholeheartedly*, *but I fail at times to put my sexual desires aside*. *I am committed to loving her but slightest inclination could trigger my urges of having sex with a male body*. *I am okay with my suffocation because I know my wife is suffocated too because she knows about my sexuality*. *She sometimes feels unwanted*. *I wish I could help her*. *I want to control my desires*. *I am spiritual*. *I want to transform my sexuality into creativity*. *I trained myself with the Art of Living* (a spiritual program), *but this did not change*. *Maybe my inner being did not want this to change*.

While both Prasen and Ashutosh were committed to loving their wives, their “unwanted” desires created a sense of confusion in their minds. Men who were married to women reported juggling between their two mutually exclusive lives and their efforts to balance the “uncontrollable and unwanted” desires.

#### Sexual behaviour in late life

Respondents such as Ashutosh discussed how sometimes their sexual desires and urges went beyond control. Watching pornography, using *Grindr*, and talking to queer men made them vulnerable to “unwanted” sexual needs. While Ashutosh mentioned his efforts to avoid such practices by not watching pornography and deleting *Grindr* from his phone, he added that seeing a “hot young guy” in public would frequently trigger the urges. Whether repeated failure to resist such impulses may lead to sexual compulsion is a question that needs further investigation by psychologists and other mental health professionals. Mohit and Pratik’s take on this was very different yet intriguing. Mohit (52y), said

*Today, my definition of making love would be very less to do with climax or orgasm; I enjoy the build-up more than the actual act. I would prefer a romantic date to sex date. That pattern has changed. That becomes a concern for some people I meet because they want to get to the act, whereas I get totally put-off. When I was in my 30s, I was okay with anything then*.

Pratik (60y), discussed the difference he observed between his gay and heterosexual peers’ sexual behaviour in late life. He said

*The libido has certainly dropped. I don’t have ‘that’ craving for hooking up. Again, this is very individualistic, because our bodies are different, our priorities are different. Single men would behave differently than us. I would want something more romantic than sexual. One difference I observed between the heterosexual circle and gay circle is in their sexual behaviour. In straight couples, in India, sexual intimacy diminishes with age, once the wife hits menopause, if I can put it right. But in the case of our community, having a partner or not, the craving for sexual intimacy stays alive for longer. It’s still a priority for men over 45. Sex is not what bonds the heterosexuals together, but I guess sex plays a very important role for gay men*.

This view reinforces the importance of sexual relations in later life among queer men and suggests that there is a perception of enhanced inclination towards sexual intimacies among the homosexual men than the heterosexual counterparts.

## Discussion

This mixed-methods study of 207 older queer men provides a rare insight into the lives of this under-researched population in India. Our results suggest that this population may be at risk of developing loneliness, depression, and sexual compulsivity due to the discrimination as a result of their doubly disadvantaged identity: sexual minority status and old age. A majority of the respondents reported facing discrimination related to their age in the form of prejudiced views and actions, which were found to have a negative impact on their mental health, especially when they lacked social support or economic resources. We unpacked the social structuring of internalized homophobia and found that it notably worsened the mental health of this population. Older gay men married to women were found to be more vulnerable to loneliness and depressive symptoms because of their “helpless” situation which sustained over years. Optimism and stress resilience emerged as key attributes among those successfully negotiating loneliness and depressive symptoms.

Several respondents in the qualitative interviews reported facing ageist comments, abuse, and other discriminatory behaviours on social media and dating apps. Since these sites were frequently the only ways for older queer men to develop social relations within the community, negligence in such spaces might lead to isolation. Such discriminatory events may also give rise to a fear of ageing which was associated with higher levels of loneliness in this study. These findings from the qualitative and quantitative strands align with what Shiovitz et al [47] argued about the experiences of ageism in different places, which restricted social opportunities for older adults, consequently limiting social engagement and elevating late-life loneliness.

Greater internalized homophobia was related to higher levels of loneliness and depressive symptoms in our quantitative study. Previous studies found that internalized homophobia significantly predicted depressive symptoms in samples of older homosexual adults in the Netherlands [6] and in the US [7]. Our qualitative findings helped us further explore this relationship. Internalized homophobia which developed in many of our participants at a very young age isolated them from their true selves for a long time. Such isolation could deprive a queer person from living an authentic life. They might refrain from getting in touch with people from the community due to the fear of escalating the “sin.” This isolation could also suppress their sexual desires and restrict them from interacting with other people leading to sense of loneliness. The qualitative understanding of internalized homophobia in this study, see for instance the narrative of Sumit, supported the claim by Meyer [8] that internalized homophobia stems from the heterosexist social attitudes in the environment that one lives in. Jensen [48] suggested that the influence of heterosexist norms influences one’s acceptance of his/her/their sexuality and restricts him/her/them from labelling same-sex attraction until late adulthood or midlife as in the case of our participants. Thoits [15] suggested that this “self-stigmatization” that one develops in early life may still harm the sexual minorities by the imposition of negative social beliefs about themselves even in the absence of any negative events in life. This corroborates with our study findings.

Higher levels of loneliness and depressive symptoms predicted higher sexual compulsivity among the older queer population in our survey. Quam [49] found in the US that older queer men were often (mis)understood as oversexed. The study by Kathryn, et al [50] concluded that a greater feeling of loneliness was associated with higher number of sexual partners among the sexual minorities in the US. However, our qualitative inquiry revealed a mixed picture. While one participant who was passing a heterosexual marriage mentioned about the slightest inclination towards a gay male body triggering his sexual compulsion, of a few of the participants shared about the decline in their sexual urges with age. The existence of suffocation and depression in the passing gay participants was reflected in their narratives. Frequently deleting *Grindr* and trying to push away sexual desires by alienating themselves from the community was a strategy many such participants adopted to keep their mind focused on their wives and family. Such isolation from the community made them further lonely and gave rise to a sense of desperation which triggered urges of sexual intimacy upon a “gentle imagination of homosexual act.” Notably, almost 70% of the older queer men married to women in our survey were classified as having a higher level of loneliness and were also the biggest group (37.14%) of older adults having higher sexual compulsivity symptoms. Thus, these results suggest that older queer men who were married to women were prone to more sexual compulsivity symptoms than those who were single or were in romantic relationships with same-sex partners. Constant juggles between the two lives that a passing homosexual man lives could give rise to complications in understanding and satisfying their desires. This highlights an ongoing tension between morality, sexuality, and the “modern” self in emergent consumerist cultures [51] which may limit the exploration of sexual authenticity of these men.

Moreover, the qualitative findings suggested the centrality of sex in the community. A lack of understanding of the spectrum of sexual orientation among the community members, as well as others, might have limited the queer men’s idea of homosexuality to be primarily as an act of sex. This could be compounded by the long absence of a legal framework and the history of criminalisation of homosexuality in India, which allowed only for secretive physical expression of their sexuality as opposed to leading a relationship-based life while fulfilling widely practised social norms.

On the other hand, social ties, social embeddedness, and social engagement were tapped by participants while addressing their loneliness, a finding corroborated by previous studies among older queer populations in the West [20, 52]. For many participants in this study, social network, involvement with community members, and the support thus induced, reduced loneliness, and also compensated for the need of a life partner at late-life. Many believed that a strong social network would act as social security at an older age. It has been previously recognized that safe public spaces, such as the one frequented by several participants (*Seenagers*), may provide opportunities for social support, collective identification, and coping [19], aligning with the stress ameliorating factors identified in Meyer et al.[53]. This “family of choice” helped several participants in specific adjustment processes because they relied on them during times of need. However, this relationship of social support with loneliness and depressive symptoms was not supported by our quantitative results. One reason that may explain these results could be the limited operationalization of the variable measuring perceived social support. It did not measure the quality or the quantity of social network but emphasized the involvement in any support group and the perception of how important social groups were to the respondents’ happiness.

With access to resources such as a wide social network of health professionals, LGB activists, counsellors, and lucrative hobbies, participants described how their lifestyle reduced loneliness and helped cope with any sadness. On the other hand, respondents with limited material resources depending on their family for financial support likely had limited ways to address loneliness. Most of the affluent respondents of the qualitative study either lived alone (a luxury in Mumbai) or with their same-sex partners. They were content and reported mild to no loneliness. This aligns with the findings of Kim & Fredriksen-Goldsen [6] that older queer men living with their same-sex partners were less lonely than those living alone or with others.

Acceptance was a major theme in staying happy at a later life not only in this study but also in many previous studies such as Fogg [54] and Tate et al. [55]. But acceptance of one’s sexuality and old age in this study mostly remained limited to participants in the higher economic stratum. Both the qualitative and quantitative findings suggested that only the affluent stratum of the older queer men managed to bypass the adverse consequences of loneliness and depression. Respondents who attended pride marches, queer social meetings, and actively took part in LGB events were mostly from this stratum. Thus, with financial stability and security older queer men appeared to gain an enhanced ability to express their sexuality.

Surprisingly, relationship status was not associated with mental health. This deviates from the results of D’Augelli, Grossman, Hershberger, & O’Connell [26] which suggested that gay men with same-sex partners were less likely to be lonely than those who were single. One explanation for this could be our operationalization of the relationship status variable. The survey item about relationship status elicited a wide variety of options such as “married to a man,” “married to a woman,” “same-sex partnered,” and “abusive relationship” but this was collapsed into a binary of single or not. This operationalization possibly failed to capture the nuances of relationship status thus limiting our ability to link it with mental health. Notably, many participants of the qualitative study perceived romantic relationships to be a social support in later life. This aligns with the findings of a few Asian studies such as the one by Chou [56] in China. It suggested that having a partner reduces the risk of rejection while coming out, even though in our study many respondents adhered to the claim that a larger social network in the form of “family of choice” was much more vital than a romantic relationship.

### Limitations and strengths

There are several limitations of this study, which need to be borne in mind while interpreting our findings. Reverse causation could be a major limitation because those respondents who were depressed might remember greater numbers of discriminatory events or even perceive non-discriminatory events as discriminatory. However, we are confident that this limitation does not completely explain our findings because our quantitative results align with our in-depth qualitative study. During discussions, AJS constantly monitored the respondents’ body language and dug deeper with probes, which gave us a comprehensive picture of how events unfolded.

One of the major drawbacks of the qualitative interviews is that the majority of the sample belonged to the middle and upper-middle-class section, who lived in urban Mumbai. The homogeneity in these class identities might have skewed the study findings. On the other hand, the quantitative study sample was diverse in terms of socioeconomic factors.

The use of online recruitment for participants’ convenience and efficiency may have limited the sample to those who used the internet. The pool of respondents who participated in the study were mostly those active participants on the Facebook queer groups (as the advertisement was posted on those groups), which biased the sample towards a more socially engaged group, who are likely to be more comfortable with their sexual identity. They might have suffered fewer discriminatory instances and have more resources in terms of social support. On the other hand, our data suggest that most ageism experiences were reported to be on such online platforms.

Another limitation is our use of self-reported depressive symptoms instead of a clinical interview which would have yielded a diagnosis of depression. We have used the self-reported GDS-15 scale because it was not feasible to conduct clinical interviews given the online method of data collection due to the inaccessibility of this study population. However, we argue that studying depressive symptoms is also informative and our study is strengthened by the use of a validated scale.

A shorter version of the resilience coping scale was used to reduce the time in filling up the “lengthy and exhaustive” survey. This might have limited our assessment of stress resilience which could be a far more complex and multidimensional construct. The longer version could have yielded nuanced results. However, this study tried to unpack several complexities with the in-depth qualitative interviews, which give us confidence in our conclusions.

The modest sample size prevented us from examining whether constructs such as social support, LGB participation, optimism, and stress resilience moderated the relationships studied. The smaller sample size did not allow us to focus on intersectionality by looking at the multiplicative and complex effects of two or more types of stressors. However, given the difficulty in recruitment due to the stigma associated with the community, we were grateful to achieve, to our knowledge, recruiting the largest sample in a study of this kind in India.

The theoretical model utilized in this study does not include several other stressors which could have led to the outcomes under study. For instance, the model does not focus on the distal and social determinants of mental health outcomes. However, the theoretical model utilized in this study looks at several important determinants such as internalized homophobia, ageism, degree of closetedness, and fear of ageing which play a vital role in this population but are under-researched. Thus, applying this perspective can inform programs focused on the psychological wellbeing of the population.

Another limitation of this study is its cross-sectional nature, thus limiting our ability to make causal claims. Longitudinal studies are needed to understand the psychosocial processes of growing old as a queer man and to establish causality with respect to the relationships studied.

Despite these limitations, the present study adds to the very small body of work on the challenges of the older queer men in India. To our knowledge, this study has the largest sample size representing older queer population in India, especially with ample representation of groups with respect to their location, sexual orientation, educational attainment, income, and relationship status. Moreover, the mixed method approach has provided a comprehensive understanding of the relationships between stressors and their psychological outcomes.

### Implications

Our findings were consistent with Meyer’s theory of sexual minority discrimination and psychological outcomes. It could open new avenues for gerontologists, counsellors and healthcare professionals to understand the lives of older homosexual men in India, their psychological needs, make decisions, and provide their services accordingly.

These findings suggest drawing the attention of friends, partners, family, and caregivers of the sexual minority older adults, through group sessions or other means, to create awareness related to discrimination, the importance of social support, and acceptance of sexual minorities. Such measures may impact the mental health and overall well being of older queer men. The study also alerts gerontologists and public health professionals to the need for further studies on this population to unravel the complex dynamics related to their wellbeing.

Our findings also support the need of educating youth, particularly those who have recently self-identified as sexual minorities, through school curriculum and campaigns about being comfortable with their sexuality. This potentially reduces the development of internalized homophobia that could have a deleterious effect at a later age.

The study advocates for access to social spaces for the older queer men to interact, such as *Seenagers*, to build community support which could buffer stress to a large extent.

Lastly, this study also calls the attention of mental health policymakers towards designing inclusive policies addressing the concerns of older sexual minorities in India. The stigma attached to the sexual minority identity is frequently a barrier to health care access, which at times of acute loneliness or depression could have severe consequences. Any additional age-related stigma hinders a sexual minority individual further in taking advantage of the health care services.

## Conclusion

Older queer men in India appear to be complex individuals with doubly disadvantaged identities vulnerable to adverse mental health outcomes. This study reinforces the call for more socially and culturally sensitive practices for eliminating discrimination related to their identities in order to boost their coping. Albeit from a relatively urban area with an awareness of homosexuality, our findings still call for policies to address the stigma and the consequent mental health issues in this population. The current mental health policies lack inclusivity thus perpetuating the deafening silence regarding the needs of this marginalized community.

## Supporting information

S1 Checklist Coreq32. (DOCX)

S1 Data Questionnaire for the qualitative component. (DOCX)

S2 Data Questionnaire for the quantitative component (online survey). (DOCX)
