# Supplementary material for: Psychological wellbeing of middle-aged and older queer men in India: A mixed-methods approach
Source: PLoS One. 2020 Mar 12;15(3):e0229893. doi: 10.1371/journal.pone.0229893 (PMC7067389; doi:10.1371/journal.pone.0229893)
Supplement: S1 Checklist — (DOCX) [file pone.0229893.s001.docx]

| COREQ-32 ITEM CHECKLIST FOR QUALITATIVE PHASE | | | |
| --- | --- | --- | --- |
| **No** | **Item** | **Guide questions/description** | **Responses** |
| **Domain 1: Research team and reflexivity** |  |  |  |
| Personal Characteristics |  |  |  |
| 1. | Interviewer/facilitator | Which author/s conducted the interview or focus group? | Sharma, A. J. (male) |
| 2. | Credentials | What were the researcher's credentials? *E.g. PhD, MD* | At the time of data collection, Sharma, A. J. was a graduate student at IIT Gandhinagar with a previous master’s degree. |
| 3. | Occupation | **Simply state in the author contributions section that the data was collected as part of your doctoral work.** | The data was collected as a part of Sharma, A.J.'s master’s dissertation research at IIT Gandhinagar. |
| 4. | Gender | Was the researcher male or female? | Sharma, A. J. is a male. |
| 5. | Experience and training | What experience or training did the researcher have? | At the time of data collection, Sharma, A. J. was new to social science research. He had taken several courses such as research methods in social sciences to acquaint himself with the skills needed for data collection and analysis. He had previously conducted semi-structured interviews for a different project on a sensitive topic (understanding stigma associated with HIV infected and affected children). Prior to commencing this study Sharma, A. J. Participated in mock interviews with his supervisor, Subramanyam, M.A. |
| Relationship with participants |  |  |  |
| 6. | Relationship established | Was a relationship established prior to study commencement? | Prior to commencing the study, researcher had contacted one key person at the study site, Mumbai. For the qualitative study, this key contact (who also is one of the participants) introduced the researcher to the other potential participants. The researcher was in touch with the participants over the phone and text (WhatsApp) and planned the interviews accordingly. |
| 7. | Participant knowledge of the interviewer | What did the participants know about the researcher? e*.g. personal goals, reasons for doing the research* | Prior to collecting the data, Sharma, A. J. ensured that he introduced himself to the participants as a graduate student engaged in a research project and added details such as the geographical area he belonged to, the university where he studied, and his motivation for this study. He clarified about the steps and phases in this study and the ethical approvals received. All the participants were provided the opportunity to ask any questions or concerns if they had any. Only after receiving consent from the participants, the data were collected. |
| 8. | Interviewer characteristics | What characteristics were reported about the interviewer/facilitator? e.g. *Bias, assumptions, reasons and interests in the research topic* |  |
| **Domain 2: study design** |  |  |  |
| Theoretical framework |  |  |  |
| 9. | Methodological orientation and theory | What methodological orientation was stated to underpin the study? *e.g. grounded theory, discourse analysis, ethnography, phenomenology, content analysis* | The study was guided by the Meyer’s minority stress model (2003) which informed the collection, thematic analysis, and presentation of qualitative (and the quantitative) data in this study. The data collected and analysis was carried out simultaneously and were mixed during interpretation as suggested by Creswell et al. (2003) |
| Participant selection |  |  |  |
| 10. | Sampling | How were participants selected? *e.g. purposive, convenience, consecutive, snowball* | A key contact in Mumbai (a social acquaintance), who is also one of the participants of the study, helped in identifying and recruiting participants for the interviews and FGDs in the qualitative phase of the study. Purposive and snowball sampling were both used to select participants. The researcher approached the participants over the phone and text (WhatsApp) and planned the interviews accordingly. There were total 35 semi-structured interviews (5 in preliminary study and 30 in the qualitative final study) and one FGD (7) of queer men in Mumbai (aged 40 and above). Although all the interviews were audio-taped, few interviews were not in order to respect participants' reservations regarding their opinions being audio-recorded. Some participants also chose not to answer a few questions when they felt uncomfortable. |
| 11. | Method of approach | How were participants approached? e*.g. face-to-face, telephone, mail, email* |  |
| 12. | Sample size | How many participants were in the study? |  |
| 13. | Non-participation | How many people refused to participate or dropped out? Reasons? |  |
| Setting |  |  |  |
| 14. | Setting of data collection | Where was the data collected? e*.g. home, clinic, workplace* | Semi-structured interviews and focus-group discussions took place either at participants' homes, or in public spaces that participants were comfortable with and ensured privacy. When the interviews were carried out outdoors (in cafes or other public spaces), there were other people present, but the participants’ comfort and privacy was not compromised. |
| 15. | Presence of non-participants | Was anyone else present besides the participants and researchers? |  |
| 16. | Description of sample | What are the important characteristics of the sample? *e.g. demographic data, date* | Since this study focused on middle-aged and older queer men’s understanding and experiences regarding their ageing and dealing with their sexuality, we tried to include participants from diverse social settings. But because the study was conducted in better-off neighborhoods of Mumbai, most of the participants belonged to the upper economic stratum. |
| Data collection |  |  |  |
| 17. | Interview guide | Were questions, prompts, guides provided by the authors? Was it pilot tested? | Interview guide was developed by the authors and reviewed by an external expert, an assistant professor at IIT Gandhinagar. Moreover, the IITGN institutional ethics committee reviewed this guide. We pilot tested it and modified the interview guide for appropriate wording and effective ordering based on the initial few interviews. |
| 18. | Repeat interviews | Were repeat interviews carried out? If yes, how many? | Six repeat interviews were carried out for respondent validation. |
| 19. | Audio/visual recording | Did the research use audio or visual recording to collect the data? | The researcher took verbal consent from all the participants and audio-taped the conversations whenever consent for the same was provided. |
| 20. | Field notes | Were field notes made during and/or after the interview or focus group? | Field notes were taken both during and after the interviews by the interviewer. |
| 21. | Duration | What was the duration of the interviews or focus group? | The interviews lasted from 40 minutes to 1.5 hours, while, the FGD lasted for around 50 minutes. |
| 22. | Data saturation | Was data saturation discussed? | Yes, we mention that we reached saturation around our 20^th^ interview. However, we increased the interviews to 30 participants to make the sample more diverse. |
| 23. | Transcripts returned | Were transcripts returned to participants for comment and/or correction? | No, transcripts were not returned to participants for comment and/or correction. |
| **Domain 3: analysis and findings** |  |  |  |
| Data analysis |  |  |  |
| 24. | Number of data coders | How many data coders coded the data? | One. |
| 25. | Description of the coding tree | Did authors provide a description of the coding tree? | All participants in the study were asked to describe their understanding, various observations and experiences regarding their ageing and sexuality and how they faced/did not face discrimination. The responses to these questions constituted the majority of the data that was analyzed in this study. The coder (Sharma, A. J.), with his own understanding and under the supervision of Subramanyam, M.A. coded the data. This coding was verified by an external coder. |
| 26. | Derivation of themes | Were themes identified in advance or derived from the data? | Some broad areas formed the basis for developing the interview guide after the preliminary study was conducted. However, new themes that emerged during the data collection were also analyzed in the final study. |
| 27. | Software | What software, if applicable, was used to manage the data? | The analysis was done manually. |
| 28. | Participant checking | Did participants provide feedback on the findings? | Yes, the findings were discussed with six participants who provided respondent validation. |
| Reporting |  |  |  |
| 29. | Quotations presented | Were participant quotations presented to illustrate the themes / findings? Was each quotation identified? e*.g. participant number* | Yes, participants' quotes were used to illustrate the findings in the manuscript. Pseudo names along with their age were used with the quotes to identify them. |
| 30. | Data and findings consistent | Was there consistency between the data presented and the findings? | Yes |
| 31. | Clarity of major themes | Were major themes clearly presented in the findings? | Yes |
| 32. | Clarity of minor themes | Is there a description of diverse cases or discussion of minor themes? | Yes |
