# Supplementary material for: Psychological wellbeing of middle-aged and older queer men in India: A mixed-methods approach
Source: PLoS One. 2020 Mar 12;15(3):e0229893. doi: 10.1371/journal.pone.0229893 (PMC7067389; doi:10.1371/journal.pone.0229893)
Supplement: S1 Data — (DOCX) [file pone.0229893.s002.docx]

**Supplementary information 2**

**Questionnaire for the qualitative component**

Kindly note that this questionnaire was used for collecting qualitative data using semi-structured conversational style interviews from the participants after getting verbal assent from participants’. The participants were told about the study before beginning the interviews. Many follow up questions were asked depending on the flow of the conversation.

Socio-demographic information:

1. Age:
2. Education:
3. Relationship status:
4. Caste:
5. Income:
6. Living arrangements (Urban/Rural):
7. Self identified sexual orientation:

Questions on their early life

1. When and how did you first realize that your were different from other boys/men (sometimes rephrased when did you realise about your queerness)?
2. What according to you is being gay/bi?
3. How comfortable were you with your sexual orientation back then?
4. What bothered you (if at all)?
5. When and how did you reveal your sexual orientation to others? What were some of the reactions from the people? What were (are) the challenges?
6. Did disclosing your sexual orientation changed your family’s attitude towards you?
7. How often did you actively take part in sexual activities?
8. How have you identified your sexual partners?
9. Tell me about your romantic relationships? Were there any complications because of the heterosexist society? What did you do?
10. Do you think there were any negative impacts of your sexual orientation on your health? Why do you think so? What were your strategies in dealing with them?
11. How comfortable were your straight friends with your sexuality?
12. Did you ever try to fit in to the heterosexist society? How and why?

Questions on late life (present day):

1. How do you feel about being Gay/Bi today?
2. What difference do you observe in your gay lifestyle from then?
3. How often do you take part in sexual activities now?
4. Is it easier for you to come out to people now than earlier? Why do you feel so?
5. What has been the role of your family/partner throughout?
6. Have you been ever asked to get married to a woman? How did you take/handle it?
7. How much do you rely on your parents/family/friends (emotionally/financially/otherwise?
8. How do you get in touch with gay men now? What is the role of mobile dating apps and social networking sites?
9. How often do you take part in LGB activities/events?
10. Do you ever feel discriminated by your heterosexual friends/people around you because of your revealed sexuality?
11. Is there any change in your queer lifestyle after the Supreme Court verdict?
12. Do you have a queer community friend circle? What activities do you take part in together?
13. How do you look at your body at this age? What is its importance with your given sexuality? Do you feel like you have to negotiate with it?
14. Did your age ever bother you or your sexual partners?
15. How does the above 40 gay population looked upon by the younger counterparts? How do you look at ageist comments on Grindr and other social networking sites/apps?
16. What is the importance of family in queer ageing?
17. What is the importance and role of a romantic relationship in queer ageing?
18. What are your present health concerns (if any)?
19. Would you like to add anything (did I miss anything to ask)?

Thanks a lot for your time!
