# Supplementary material for: Psychological wellbeing of middle-aged and older queer men in India: A mixed-methods approach
Source: PLoS One. 2020 Mar 12;15(3):e0229893. doi: 10.1371/journal.pone.0229893 (PMC7067389; doi:10.1371/journal.pone.0229893)
Supplement: S2 Data — (DOCX) [file pone.0229893.s003.docx]

**Supplementary information 3**

**Questions for the quantitative component (online survey)**

Hello, I am Anupam Sharma, a postgraduate student from the Indian Institute of Technology, Gandhinagar. As part of a research project, I am trying to understand the everyday experience-challenges, struggles, negotiations-of being gay/bisexual/men who are attracted to men in India.

If you Indian and are 40 years or above, please fill up the answers to the corresponding questions in one go without giving a second thought. Please respond as accurately and honestly as you can. There are no right or wrong answers and these questions are designed to know what you think. Please click on your answer below. These questions are designed to understand your experiences with your sexuality and its overall impact on your life.

Please understand that your participation in this research is entirely voluntary and you can stop answering at any point. Filling up this form may take 15-25 minutes of your time. All information that you share with me during the interview will be kept entirely confidential. As an ethical researcher, I will not share these details with anyone outside the research team.

We are focused only on non-trans-cis gay/bi/men attracted to men

Thank you for your time.

Age (In Years) *

I am *

Gay

Bisexual

Confused or Questioning

Other:

Relationship Status *

Single

Married (have wife)

Married (have a husband)

In a relationship

Other:

Highest Education Qualification *

PhD

Post Graduate

Graduate

12th

10th

Primary

Other:

Living with *

Alone

Partner

Family

Friends

Other:

I am *

Closeted (no straight friend or family knows)

Out to everyone

Partially out

Other:

Where do you live? *

Rural

Urban

Other:

State of residence:

Please answer the following questions as honestly as you can.

1. I have tried to stop being attracted to men in general. *

Disagree Strongly

Disagree

Neutral

Agree

Strongly Agree

2. If someone offered me the chance to be completely heterosexual, I would accept the chance. *

Disagree Strongly

Disagree

Neutral

Agree

Strongly Agree

3. I wish I weren't gay/bisexual. *

Disagree Strongly

Disagree

Neutral

Agree

Strongly Agree

4. I feel that being gay/bisexual is a personal shortcoming for me. *

Disagree Strongly

Disagree

Neutral

Agree

Strongly Agree

5. I would like to get professional help in order to change my sexual orientation from gay/bisexual to straight. *

Disagree Strongly

Disagree

Neutral

Agree

Strongly Agree

6. I have tried to become more sexually attracted to women. *

Disagree Strongly

Disagree

Neutral

Agree

Strongly Agree

7. I often feel it best to avoid personal or social involvement with other gay/bisexual men. *

Disagree Strongly

Disagree

Neutral

Agree

Strongly Agree

8. I feel alienated from myself because of being gay/bisexual. *

Disagree Strongly

Disagree

Neutral

Agree

Strongly Agree

9. I wish that I could develop more erotic feelings about women. *

Disagree Strongly

Disagree

Neutral

Agree

Strongly Agree

10. I have a good understanding with the people around me. *

Never

Rarely

Sometimes

Often

11. I feel I lack companionship. *

Never

Rarely

Sometimes

Often

12. I feel there is no one I can turn to. *

Never

Rarely

Sometimes

Often

13. I do not feel alone. *

Never

Rarely

Sometimes

Often

14.I feel part of a group of friends. *

Never

Rarely

Sometimes

Often

15. I have a lot in common with the people around me. *

Never

Rarely

Sometimes

Often

16. I am no longer close to anyone. *

Never

Rarely

Sometimes

Often

17. My interests and ideas are not shared by those around me. *

Never

Rarely

Sometimes

Often

18. I am an outgoing person. *

Never

Rarely

Sometimes

Often

19. There are people I feel close to. *

Never

Rarely

Sometimes

Often

20. I feel left out. *

Never

Rarely

Sometimes

Often

21. My social relationships are superficial. *

Never

Rarely

Sometimes

Often

22. No one really knows me well. *

Never

Rarely

Sometimes

Often

23. I feel isolated from others. *

Never

Rarely

Sometimes

Often

24. I can find companionship when I want it. *

Never

Rarely

Sometimes

Often

25. There are people who really understand me. *

Never

Rarely

Sometimes

Often

26. I am unhappy being so withdrawn. *

Never

Rarely

Sometimes

Often

27. People are around me but not with me. *

Never

Rarely

Sometimes

Often

28. There are people I can talk to. *

Never

Rarely

Sometimes

Often

29. There are people I can turn to. *

Never

Rarely

Sometimes

Often

Please respond on a 4-point scale that ranges from 1 (not at all like me) to 4 (very much like me)

30. My sexual appetite has gotten in the way of my relationships. *

1 (Not at all like me)

2 (Not like me)

3 (Like me)

4 (Very much like me)

31. My sexual thoughts and behaviors are causing problems in my life. *

1(Not at all like me)

2 (Not like me)

3 (Like me)

4 (Very much like me)

32. My desires to have sex have disrupted my daily life. *

1 (Not at all like me)

2 (Not like me)

3 (Like me)

4 (Very much like me)

33. I sometimes fail to meet my commitments and responsibilities because of my sexual behaviors. *

1 (Not at all like me)

2 (Not like me)

3 (Like me)

4 (Very much like me)

34. I sometimes get so horny I could lose control. *

1 (Not at all like me)

2 (Not like me)

3 (Like me)

4 (Very much like me)

35. I find myself thinking about sex while at work. *

1 (Not at all like me)

2 (Not like me)

3 (Like me)

4 (Very much like me)

36. I feel that sexual thoughts and feelings are stronger than I am. *

1 (Not at all like me)

2 (Not like me)

3 (Like me)

4 (Very much like me)

37. I have to struggle to control my sexual thoughts and behavior. *

1 (Not at all like me)

2 (Not like me)

3 (Like me)

4 (Very much like me)

38. I think about sex more than I would like to. *

1 (Not at all like me)

2 (not like me)

3 (Like me)

4 (Very much like me)

39. It has been difficult for me to find sex partners who desire having sex as much as I want to. *

1 (Not at all like me)

2 (not like me)

3 (Like me)

4 (Very much like me)

Please consider how well the following statements describe your behavior and actions.

40. I look for creative ways to alter difficult situations. *

Does not describe me at all

Does not describe me

Neutral

Describes me

Describes me very well

41. Regardless of what happens to me, I believe I can control my reaction to it. *

Does not describe me at all

Does not describe me

Neutral

Describes me

Describes me very well

42. I believe I can grow in positive ways by dealing with difficult situations. *

Does not describe me at all

Does not describe me

Neutral

Describes me

Describes me very well

43. I actively look for ways to replace the losses I encounter in life. *

Does not describe me at all

Does not describe me

Neutral

Describes me

Describes me very well

44. In uncertain times, I usually expect the best *

Strongly Disagree

Disagree

Neutral

Agree

Strongly Agree

45. It’s easy for me to relax. *

Strongly Disagree

Disagree

Neutral

Agree

Strongly Agree

46. If something can go wrong for me, it will. *

Strongly Disagree

Disagree

Neutral

Agree

Strongly Agree

47. I’m always optimistic about my future. *

Strongly Disagree

Disagree

Neutral

Agree

Strongly Agree

48. I enjoy being with my friends a lot. *

Strongly Disagree

Disagree

Neutral

Agree

Strongly Agree

49. It’s important for me to keep busy. *

Strongly Disagree

Disagree

Neutral

Agree

Strongly Agree

50. I hardly ever expect things to go my way. *

Strongly Disagree

Disagree

Neutral

Agree

Strongly Agree

51. I don’t get upset too easily. *

Strongly Disagree

Disagree

Neutral

Agree

Strongly Agree

52. I rarely count on good things happening to me. *

Strongly Disagree

Disagree

Neutral

Agree

Strongly Agree

53. Overall, I expect more good things to happen to me than bad. *

Strongly Disagree

Disagree

Neutral

Agree

Strongly Agree

54. In the last year have you ever been discriminated anywhere (dating apps/real life) by someone because of your age? *

Yes

No

Dating app

Real life

Other:

55. Do you actively participate in LGBTQ events? *

Yes

No

Maybe

Other:

56. Aside from LGBT community, are you involved in other organizations, groups, clubs, or activities in your local community? *

Yes

No

Maybe

Other:

57. Do you have any fear about aging as a gay man? *

Yes

No

Maybe

Other:

58. On a scale from 1 to 10 please rate how successfully you see yourself aging. (1= Least Successful, 10= Most successful) *

59. Do you work on your body to look younger than your age? *

Yes

No

Sometimes

60. Are you basically satisfied with your life? *

Yes

No

70. Have you dropped many of your activities and interests? *

Yes

No

71. Do you feel that your life is empty? *

Yes

No

72. Do you often get bored? *

Yes

No

73. Are you in good spirits most of the time? *

Yes

No

74. Are you afraid that something bad is going to happen to you? *

Yes

No

75. Do you feel happy most of the time? *

Yes

No

76. Do you often feel helpless? *

Yes

No

77. Do you prefer to stay at home, rather than going out and doing new things? *

Yes

No

78. Do you feel you have more problems with memory than most? *

Yes

No

79. Do you think it is wonderful to be alive now? *

Yes

No

80. Do you feel pretty worthless the way you are now? *

Yes

No

81. Do you feel full of energy? *

Yes

No

82. Do you feel that your situation is hopeless? *

Yes

No

83. Do you think that most people are better off than you are? *

Yes

No

84. In general, how would you rate your health today *

Very good

Good

Moderate

Bad

Very Bad

85. On a scale from 0 to 4, how important do you think a romantic relationship/having a partner/husband is to be happy at your age? (0 being 'not at all important' and 4 being 'very important') *

86. Do you work on your body to get attention from other gay men in real or on dating apps? *

Yes

No

Sometimes

87. On a scale from 0 to 4, how happy are you using dating apps at your age? (0 being 'not at all happy' and 4 being 'very happy') *

0

1

2

3

4

Don't use dating apps

88. Do you think people take/took advantage of you because of your age? *

Yes

No

Sometimes

89. Compared to one year ago, how would you rate your health in general now? *

Much better than one year ago

Somewhat better than a year ago

About the same

Somewhat worse now than one year ago

90. How often do you have sex with men? *

Never

Rarely

Sometimes

Often

Very often

91. Are you a part of any LGBTQ support group? *

Yes (online)

Yes (offline)

Yes (offline and online both)

No

92. On a scale of 0 to 10 how beneficial do you think LGBTQ support groups (offline or online) in keeping you happy? *

93. Annual Income *

0-3 L

3-10 L

10-20 L

20-50 L

50 L +

Thank You!
